# Tumor tissue and plasma levels of AXL and GAS6 before and after tyrosine kinase inhibitor treatment in EGFR‐mutated non–small cell lung cancer

**DOI:** 10.1111/1759-7714.13166

**Published:** 2019-08-16

**Authors:** Yoshikane Nonagase, Masayuki Takeda, Koichi Azuma, Hidetoshi Hayashi, Koji Haratani, Kaoru Tanaka, Kimio Yonesaka, Hidenobu Ishii, Tomoaki Hoshino, Kazuhiko Nakagawa

**Affiliations:** ^1^ Department of Medical Oncology Kindai University Faculty of Medicine Osaka‐Sayama, Osaka Japan; ^2^ Division of Respirology, Neurology, and Rheumatology, Department of Internal Medicine Kurume University School of Medicine Kurume, Fukuoka Japan

**Keywords:** AXL, EGFR‐TKI, GAS6, liquid biopsy and non‐small cell lung cancer

## Abstract

**Background:**

Non‐small cell lung cancer (NSCLC) positive for activating mutations of the epidermal growth factor receptor (EGFR) gene is initially sensitive to EGFR‐tyrosine kinase inhibitors (TKIs) but eventually develops resistance to these drugs. Upregulation of the receptor tyrosine kinase AXL in tumor tissue has been detected in about one‐fifth of NSCLC patients with acquired resistance to EGFR‐TKIs. However, the clinical relevance of the levels of AXL and its ligand GAS6 in plasma remains unknown.

**Methods:**

Tumor tissue and plasma specimens were collected from 25 EGFR‐mutated NSCLC patients before EGFR‐TKI treatment or after treatment failure. The levels of AXL and of GAS6 mRNA in tumor tissue were evaluated by immunohistochemistry and chromogenic in situ hybridization, respectively. The plasma concentrations of AXL and GAS6 were measured with enzyme‐linked immunosorbent assays.

**Results:**

AXL expression was detected in three of 12 (25%) and nine of 19 (47%) tumor specimens obtained before and after EGFR‐TKI treatment, respectively. All tumor specimens assayed were positive for GAS6 mRNA. The median values for the plasma AXL concentration before and after EGFR TKI treatment were 1 635 and 1 460 pg/mL, respectively, and those for the plasma GAS6 concentration were 4 615 and 6 390 pg./mL, respectively. There was no significant correlation between the plasma levels of AXL or GAS6 and the corresponding expression levels in tumor tissue.

**Conclusion:**

Plasma concentrations of AXL and GAS6 do not reflect tumor expression levels, and their measurement is thus not a viable alternative to direct analysis of tumor tissue in EGFR‐mutated NSCLC.

## Introduction

Individuals with non‐small cell lung cancer (NSCLC) positive for activating mutations of the epidermal growth factor receptor (EGFR) gene are sensitive to first‐ and second‐generation EGFR–tyrosine kinase inhibitors (TKIs) such as gefitinib, erlotinib, and afatinib. However, most such tumors develop resistance to EGFR‐TKIs within 10 to 14 months of treatment initiation.^1^, ^2^, ^3^, ^4^, ^5^, ^6^ Several mechanisms of acquired resistance to EGFR‐TKIs have been identified, including the development of a secondary T790M mutation in exon 20 of EGFR, MET amplification, overexpression of hepatocyte growth factor, and activation of insulin‐like growth factor‐1 receptor signaling.^7^, ^8^ The T790M mutation of EGFR is the most common mechanism of such acquired resistance, having been detected in up to 50% of patients after failure of EGFR‐TKI therapy.^7^, ^8^, ^9^ Osimertinib is a third‐generation, irreversible EGFR‐TKI that was approved by the U.S. Food and Drug Administration in November 2015 for the treatment of individuals with metastatic NSCLC positive for the T790M mutation of EGFR after the development of resistance to first‐ or second‐generation EGFR‐TKIs. There is currently no approved targeted drug for patients with non‐T790M‐mediated resistance to these drugs.

AXL is a member of the TAM family of receptor tyrosine kinases that also includes TYRO3 and MER.^10^ Upregulation of AXL expression has been detected in ~20% of EGFR activating mutation‐positive NSCLC patients who have developed resistance to EGFR‐TKIs.^11^, ^12^ No targeted drug for AXL or its ligand, growth arrest‐specific 6 (GAS6), has been approved to date, although several investigational AXL‐TKIs, including ASP2215, DS1205, and BGB324, are under evaluation for treatment of EGFR‐mutated NSCLC patients who have acquired resistance to first‐ or second‐generation EGFR‐TKIs due to non‐T790M‐mediated mechanisms or of those with T790M‐ positive tumors who have acquired resistance to osimertinib (ClinicalTrials.gov identifiers NCT02495233, NCT03599518, and NCT02424617, respectively).

Although a rebiopsy has been required to assess the T790M mutation status of a tumor after the development of resistance to EGFR‐TKIs, this procedure is often problematic depending on the size and location of the tumor. Liquid biopsy, a noninvasive approach to the detection and analysis of tumor components such as DNA in blood, has the potential to allow detection of cancer, measurement of tumor mutation burden, and identification of drug resistance mechanisms.^13^ Indeed, it was recently approved as an alternative to rebiopsy for detection of the T790M mutation of EGFR. GAS6 and a soluble form of AXL have been detected in plasma,^14^ although the relevance of their concentrations to EGFR mutation–positive NSCLC has remained unknown. Given the investigational status of AXL inhibitors, a noninvasive method for assessment of AXL or GAS6 levels in NSCLC tumors is desirable. We have therefore now evaluated AXL and GAS6 levels in both tumor tissue and plasma as well as their relation to clinical course in EGFR‐mutated NSCLC patients before treatment with and after the development of resistance to EGFR‐TKIs.

## Methods

### Patients and specimens

Tumor tissue and plasma samples were obtained between 25 November 2011 and 31 March 2015 from EGFR‐mutated NSCLC patients before or after treatment with first‐ or second‐generation EGFR‐TKIs at Kindai University Hospital or Kurume University Hospital. The choice of EGFR‐TKI regimen was made by the treating physician. Data regarding clinicopathologic features and treatment history were extracted from a review of the medical records. Results for C‐reactive protein (CRP) were included if the plasma concentration was measured within three days of blood sampling for measurement of AXL and GAS6. Tumor response was examined by computed tomography and was evaluated according to the Response Evaluation Criteria in Solid Tumors (RECIST) version 1.1. Progression‐free survival (PFS) and overall survival (OS) were measured from the date of initiation of EGFR‐TKI treatment until the date of disease progression or death, respectively. Postprogression survival (PPS) was calculated as OS minus PFS. This study was approved by the Institutional Review Board of each institution, and patients provided written informed consent.

### Immunohistochemistry (IHC) for AXL and chromogenic in situ hybridization (CISH) for GAS6 mRNA in tissue specimens

Formalin‐fixed, paraffin‐embedded sections of tumor tissue (thickness of 4 μm) were prepared and subjected to IHC with a rabbit monoclonal antibody to AXL (#8661, Cell Signaling Technology) and a Leica Bond RX Autostainer. The AXL H‐score (range of 0–300) was calculated by multiplying the percentage of cells positive for AXL staining and the intensity of AXL staining. A tumor was defined as positive for AXL expression if the AXL H‐score was ≥1. The tissue sections were also subjected to CISH for GAS6 mRNA with a GAS6 RNAscope Probe (No. 427818, Advanced Cell Diagnostics) and a Leica Bond RX Autostainer. Classification and quantification of CISH signals for GAS6 mRNA were performed with Computational Tissue Analysis software (Flagship Biosciences), and the percentage of GAS6 mRNA–positive tumor cells was determined. The H‐score for GAS6 mRNA (range of 0–300) was calculated with the following formula: H‐score = ([% at 1+] × 1) + ([% at 2+] × 2) + ([% at 3+] × 3), where % refers to the percentage of tumor cells at each intensity level, 1+ refers to 1 to 3 CISH dots per cell, 2+ to 4 to 6 CISH dots per cell, and 3+ to ≥7 CISH dots per cell. Tumors were defined as positive for GAS6 mRNA if the H‐score was ≥1.

### ELISAs for AXL and GAS6 in plasma specimens

Blood specimens were collected into anticoagulant‐treated tubes and centrifuged for 10 minutes at 2 000 × *g* in a refrigerated centrifuge. The isolated plasma was stored at −80°C until analysis. The concentrations of AXL and GAS6 in plasma were determined with enzyme‐linked immunosorbent assay (ELISA) kits (DY154 and DY885B, respectively, R&D Systems). Each assay was performed in duplicate.

### Statistical analysis

The relation between two parameters was evaluated with the Spearman rank correlation test, with an absolute value for the correlation coefficient (*r*) of 0–0.39 being regarded as weak, 0.40–0.69 as moderate, and 0.70–1.0 as strong.^15^ Statistical analysis was performed with the use of GraphPad Prism 5.0 software (GraphPad Software).

## Results

### Clinical characteristics of the study patients

Twenty‐five patients with NSCLC positive for EGFR activating mutations who had tissue and plasma specimens collected before treatment with EGFR‐TKIs or after the development of resistance to these drugs were enrolled in the study (Table 1). The patients comprised eight (32%) men and 17 (68%) women and had a median age of 66 years. Twenty‐two (88%) patients had an Eastern Cooperative Oncology Group performance status (PS) of 0 or 1, and three (12%) a PS of two, at the onset of initial EGFR‐TKI therapy. Twelve (48%) patients were never‐smokers, nine (36%) were ever‐smokers (former or current), and four(16%) were of unknown smoking status. A total of 24 (96%) patients were diagnosed with adenocarcinoma, and one (4%) with squamous cell carcinoma. Seventeen (68%) and eight (32%) patients had an exon‐19 deletion or L858R point mutation in exon 21 as the EGFR activating mutation at initial diagnosis, and two patients (8%) were also positive for the T790M point mutation in exon 20 before the onset of initial EGFR‐TKI treatment. The initial EGFR‐TKI administered was gefitinib in 17 (68%) patients, erlotinib in four (16%), afatinib in two (8%), and dacomitinib in two (8%). A total of 21 (84%) patients showed a partial response (PR), one (4%) stable disease (SD), and three (12%) progressive disease (PD) as the best response to initial EGFR‐TKI treatment.

**Table 1:** AXL and GAS6 expression and characteristics of the patients

						EGFR			T790M^*1^	AXL		sAXL		GAS6		sGAS6	
ID	Sex	Age	PS	Smoke	Histology	Pre‐	EGFR‐TKI	Response	Post	Pre‐	Post	Pre‐	Post	Pre‐	Post	Pre‐	Post
1	F	70	1	Never	AD	DEL19^*2^	Gefitinib	PD	+^*3^	N/A^*5^	1	N/A	1200	N/A	16.2	N/A	6920
2	F	67	0	Ever^*6^	SQ	L858R^*4^	Gefitinib	PD	+^*3^	N/A	0	N/A	1910	N/A	18.8	N/A	9790
3	F	65	1	Ever	AD	L858R	Gefitinib	PR	+	N/A	50	N/A	984	N/A	4.5	N/A	4930
4	F	74	1	Never	AD	L858R	Gefitinib	PR	−	0	1	N/A	1720	N/A	N/A	N/A	6070
5	F	52	1	Ever	AD	Del19	Gefitinib	PR	+	N/A	0	N/A	1020	N/A	9.2	N/A	4400
6	M	42	1	Never	AD	Del19	Gefitinib	PR	−	0	0	N/A	1450	12.2	24.5	N/A	7310
7	F	57	0	Never	AD	Del19	Gefitinib	PR	−	N/A	2	N/A	2050	N/A	9.3	N/A	6720
8	F	72	1	Never	AD	Del19	Gefitinib	PR	+	N/A	0	N/A	1320	N/A	13.4	N/A	6390
9	F	55	0	Never	AD	Del19	Gefitinib	PR	+	N/A	0	N/A	1990	N/A	4.1	N/A	5270
10	F	62	1	Never	AD	Del19	Gefitinib	PR	+	N/A	0	N/A	1620	N/A	20.2	N/A	8600
11	M	74	2	UNK	AD	Del19	Gefitinib	PR	−	N/A	0	N/A	1570	N/A	6.5	N/A	8590
12	M	69	1	Ever	AD	L858R	Erlotinib	PR	−	N/A	0	N/A	1570	N/A	11.1	N/A	3830
13	M	55	1	Ever	AD	Del19	Gefitinib	PR	+	N/A	10	N/A	1450	N/A	28.3	N/A	5950
14	F	53	1	UNK	AD	Del19	Gefitinib	PR	−	N/A	0	N/A	1760	N/A	7.9	N/A	7900
15	M	60	2	Ever	AD	Del19	Erlotinib	PR	−	N/A	1	N/A	1460	N/A	12.2	N/A	5690
16	M	60	1	Never	AD	L858R	Dacomitinib	PR	N/A	10	N/A	1590	N/A	14.9	N/A	6670	N/A
17	F	72	0	Ever	AD	L858R	Gefitinib	PR	N/A	0	N/A	1460	N/A	13.2	N/A	4480	N/A
18	F	71	1	Never	AD	Del19	Dacomitinib	PR	N/A	0	N/A	2090	N/A	5.6	N/A	6330	N/A
19	F	79	2	Ever	AD	Del19	Erlotinib	PR	N/A	0	N/A	1660	N/A	17.1	N/A	3760	N/A
20	F	70	0	Never	AD	L858R	Erlotinib	PR	N/A	0	N/A	1610	N/A	12.9	N/A	4240	N/A
21	F	66	1	Never	AD	Del19	Gefitinib	PR	+	2	110	1870	N/A	3.7	10.3	4610	N/A
22	M	55	1	Ever	AD	Del19	Afatinib	PR	+	2	2	1830	N/A	6.3	6.2	4370	N/A
23	M	76	1	Never	AD	Del19	Gefitinib	SD	N/A	0	N/A	1370	N/A	17.7	N/A	4380	N/A
24	F	80	1	UNK	AD	Del19	Gefitinib	PD	N/A	0	0	1660	1410	35.1	10.2	4660	6860
25	F	65	0	UNK	AD	L858R	Afatinib	PR	−	0	35	1190	1230	3.0	4.3	4620	4110

Age, Smoke, PS was evaluated at initiation of initial EGFR‐TKI. Pre‐, before treatment with EGFR‐TKI; post, after resistance to EGFR‐TKI.

*1 T790M mutation, *2 exon19 deletion, *3 de novo T790M mutation, *4 exon 21 L858R mutation, *5 not appreciated, *6 former and current smoker.

AD, Adeno; AXL, AXL H‐score; GAS6, percentage of GAS6‐positive cells; ID, patient number; PD, progression disease; sPR, partial response; sAXL, plasma AXL (pg/ml); SD, stable disease; sGAS6, plasma GAS6 (pg/ml); SQ, squamous; UNK, unknown.

### AXL protein and GAS6 mRNA levels in pre‐ and posttreatment tumor tissue

**Figure 1:**
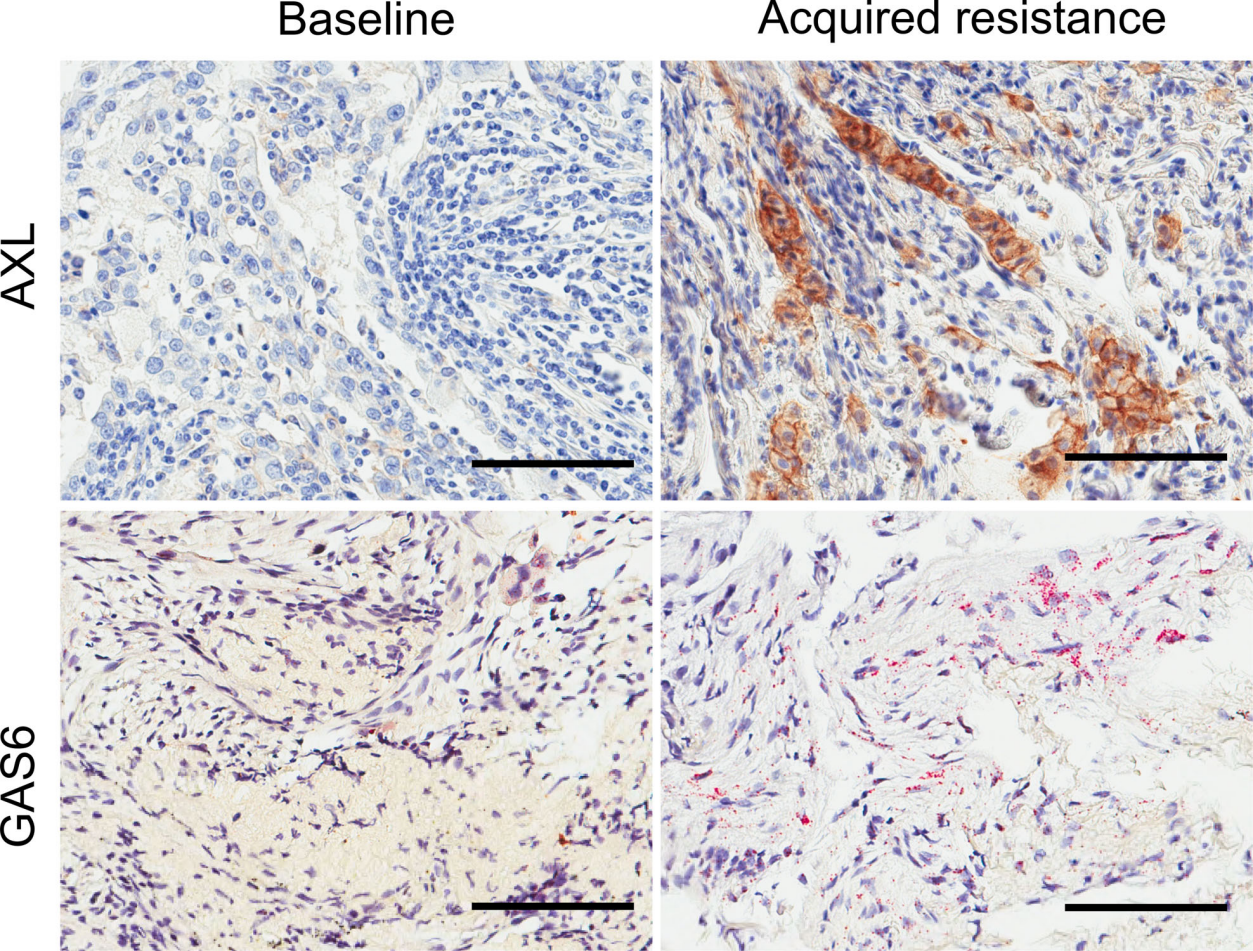
Immunohistochemistry for AXL and chromogenic in situ hybridization for GAS6 mRNA in tumor tissue. Representative images for AXL (patient ID No. 21) and GAS6 mRNA (patient ID No. 6) staining in NSCLC tissue obtained before EGFR‐TKI treatment and after the development of drug resistance. Scale bars, 100 μm.

Among the 25 study patients, 12 (48%) individuals had tissue or plasma specimens collected before EGFR‐TKI treatment, whereas 19 (76%) patients had such specimens collected after the development of EGFR‐TKI resistance (Table 1). Six (24%) patients had tissue specimens obtained before and after EGFR‐TKI treatment. IHC of AXL expression in tumor specimens revealed that three of 12 (25%) pretreatment specimens and nine of 19 (47%) post‐treatment specimens were positive for AXL expression (H‐score of ≥1). Although no patient showed high AXL expression (H‐score of >10) in pretreatment tumor specimens, three patients showed high AXL expression in post‐treatment tumor specimens (Fig 1). CISH revealed that all pre‐ and post‐treatment tissue specimens examined were positive for GAS6 mRNA (Fig 1). There was no significant correlation between the AXL H‐score and the GAS6 mRNA H‐score (*r* = −0.15) in all 29 pre‐ or posttreatment tissue specimens with data for both scores (Fig S1). Among the six patients with pre‐ and post‐treatment tissue specimens assayed for AXL, 3 (50%) individuals (ID Nos. 4, 21, and 25) showed upregulation of AXL after failure of EGFR‐TKI therapy. Among the five patients with such specimens assayed for GAS6 mRNA, 3 (60%) individuals (ID Nos. 6, 21, and 25) showed upregulation of GAS6 mRNA after treatment failure (Table 1).

AXL positivity in tumor tissue after the development of EGFR‐TKI resistance was 50% (5/10) for T790M‐positive specimens and 50% (4/8) for T790M‐negative specimens. Among the three patients who showed upregulation of AXL expression after resistance development, one individual (ID No. 21) was T790M positive and the remaining two were T790M negative.

### AXL and GAS6 levels in pre‐ and posttreatment plasma samples

**Figure 2:**
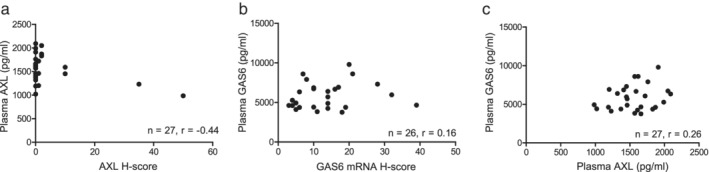
Relations Between AXL and GAS6 Levels in tumor tissue and plasma. (**a**) Relation between the plasma concentration and tissue H‐score for AXL. (**b**) Relation between the plasma concentration of GAS6 and the tissue H‐score for GAS6 mRNA. (**c**) Relation between the plasma concentrations of GAS6 and AXL. Spearman's correlation coefficient (*r*) is indicated for each plot.

Among the 25 study patients, both AXL and GAS6 were assayed in plasma for 10 patients before EGFR‐TKI treatment and for 17 patients after the development of EGFR‐TKI resistance. Two patients had paired plasma specimens from before and after EGFR‐TKI treatment that were assayed for both AXL and GAS6. The median values for the plasma concentration of AXL before and after EGFR‐TKI treatment were 1 635 pg./ml (range, 1190–2 090 pg./mL) and 1460 pg./mL (range, 984–2 050 pg./mL), respectively. The median values for the plasma concentration of GAS6 before and after EGFR‐TKI treatment were 4 615 pg./mL (range, 3760–6670 pg./mL) and 6 390 pg./mL (range, 3830–9790 pg./mL), respectively (Table 1). There was no significant correlation between tissue and plasma levels of AXL or GAS6 (protein or mRNA) or between the plasma concentrations of AXL and GAS6 (Fig 2). There was a moderate correlation between the plasma concentration of CRP and that of either AXL or GAS6 (Fig S2).

### Relation of AXL or GAS6 levels to PFS, OS, and PPS

**Figure 3:**
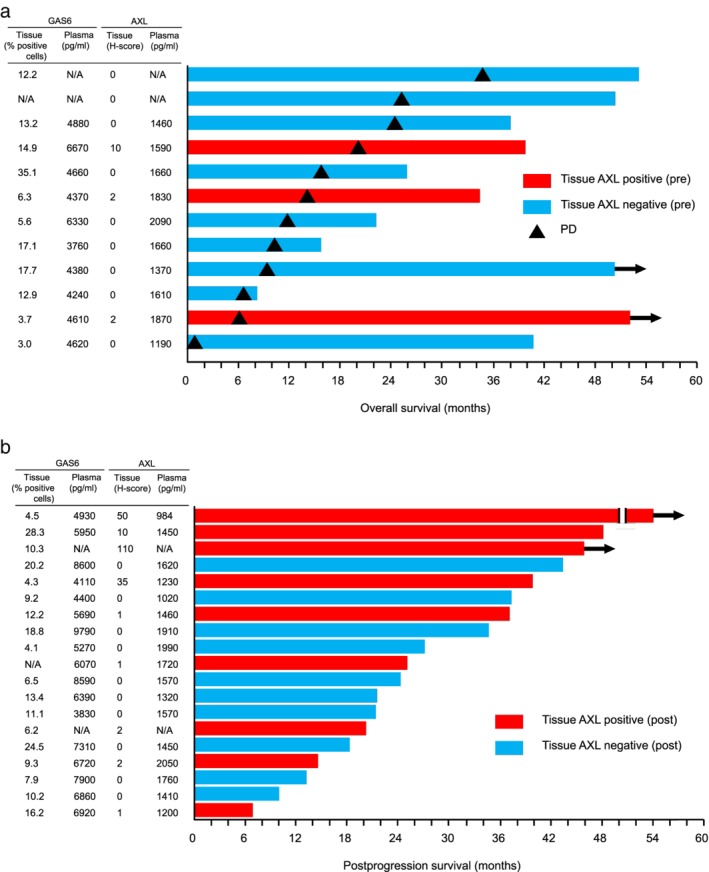
Swimmers plots of survival according to AXL expression in tumor tissue. (**a**) Swimmer plot of overall survival for patients according to the H‐score for AXL expression in tumor tissue before EGFR‐TKI treatment (pre). Red and blue bars indicate AXL‐positive (H‐score of ≥1) and AXL‐negative tumors, respectively, and bar length denotes overall survival after initiation of EGFR‐TKI therapy. Triangles indicate disease progression (PD) during initial EGFR‐TKI therapy, and arrows show the patient was alive at last follow‐up. (**b**) Swimmer plot of postprogression survival for patients according to the H‐score for AXL expression in tumor tissue after the development of resistance to initial EGFR‐TKI therapy (post). Bar length indicates postprogression survival after the development of EGFR‐TKI resistance. N/A, not available.

Finally, we examined the relation of AXL or GAS6 levels before or after EGFR‐TKI treatment to survival by constructing swimmer plots (Fig 3). The median PFS of patients with tumor tissue positive or negative for AXL expression before EGFR‐TKI treatment was 14.3 and 11.9 months, respectively, whereas the corresponding values for OS were 40.5 and 32.1 months, respectively (Fig 3(a)). No tendency toward prolonged survival was apparent for patients with tumors found to be negative for AXL expression either before EGFR‐TKI treatment (Fig 3(a)) or after treatment failure (Fig 3(b)).

## Discussion

Resistance to EGFR‐TKIs in NSCLC remains an important clinical problem. The T790M mutation of EGFR has been detected in up to 50% of NSCLC patients who develop resistance to first‐ or second‐generation EGFR‐TKIs.^7^, ^8^, ^9^ Osimertinib is a third‐generation EGFR‐TKI that shows efficacy for T790M‐positive NSCLC and can also be used in clinical practice as a second‐line treatment if the T790M mutation is detected by liquid biopsy in patients for whom tissue rebiopsy is problematic.^16^ In contrast, an effective targeted drug for T790M‐negative NSCLC resistant to EGFR‐TKIs has not yet been approved. Mechanisms of resistance to EGFR‐TKIs other than T790M include MET amplification, overexpression of hepatocyte growth factor, activation of insulin‐like growth factor–1 receptor signaling, and up‐regulation of AXL, and several agents that target these mechanisms are under development (ClinicalTrials.gov identifiers NCT02499614, NCT02495233, NCT03599518, and NCT02424617). Given the clinical benefit of detection of the T790M mutation of EGFR in plasma, detection of the operation of these other resistance mechanisms by liquid biopsy is also desirable. In the present study, we measured the plasma concentrations of AXL and its ligand GAS6 to examine whether they might reflect the corresponding expression levels in tumor tissue. However, there was no significant correlation between tissue and plasma levels of AXL or GAS6. With regard to this discordance, it has not been clearly shown that AXL present in plasma actually originates from tumor cells.^17^ AXL and GAS6 are expressed not only in tumor cells but also in various other cell types including fibroblasts as well as endothelial, smooth muscle, and bone marrow cells.^18^ In addition, the circulating concentrations of AXL and GAS6 are increased in individuals with inflammatory diseases such as liver cirrhosis, multiple sclerosis, critical limb ischemia, and sepsis, suggesting that they might be correlated with markers of inflammation such as CRP.^14^, ^19^, ^20^ In the present study, there was a moderate correlation between the circulating level of CRP and those of AXL and GAS6, suggesting that AXL or GAS6 in the blood of NCSLC patients might originate to a greater extent from cells related to inflammation than from cancer cells.^17^

Upregulation of AXL expression in tumor tissue was apparent after the development of EGFR‐TKI resistance in patients of the present study. The percentage of AXL‐positive patients was thus 25% (3/12) before EGFR‐TKI treatment and 47% (9/19) after treatment failure, consistent with the previous finding that ~20% of patients showed upregulation of AXL after the development of EGFR‐TKI resistance.^11^, ^12^ Given that AXL has also been shown to confer de novo resistance to EGFR‐TKIs,^21^ and a recent preclinical study has shown that AXL expression was increased after resistance to Osimertinib in EGFR mutant cells,^22^ the combination of an EGFR‐TKI and an AXL inhibitor may be warranted for EGFR‐TKI–naïve patients and patients with acquired resistance to EGFR‐TKIs.

AXL expression was found to be a poor prognostic marker for OS and disease‐free survival after complete resection of early‐stage NSCLC.^23^ In advanced NSCLC, PFS for EGFR‐TKI treatment tended to be shorter in patients with AXL expression scores of 3+ than in those with scores of 0 to 2+, although the difference was not statistically significant. Another study investigating the impact of AXL on EGFR‐TKI efficacy has shown that activation of AXL was associated with poor outcome of EGFR‐TKIs.^24^ However, we did not detect a tendency for OS or PFS to be shorter in EGFR‐mutated NSCLC patients with high levels of AXL or GAS6 in pretreatment tissue or plasma specimens. A previous study found that six of 12 NSCLC patients with a high AXL expression level in tumor tissue harbored EGFR activating mutation, and one of those six patients was treated with gefitinib and responded well.^25^ Together, these various observations indicate that the impact of high AXL expression on survival in EGRF‐TKI–naïve patients with EGFR‐mutated NSCLC needs further evaluation.

A limitation of the present study was the small number of patients, especially of those with paired specimens available. However, we have shown that the concentrations of AXL and GAS6 in plasma were not correlated with the corresponding expression levels in tumor tissue of NSCLC patients harboring EGFR activating mutations. Measurement of circulating AXL or GAS6 thus does not appear to be a viable alternative to direct determination of their expression levels in tumor tissue, at least for this patient population.

## Disclosure

Dr. Takeda received honoraria from Novartis Pharma, ONO Pharmaceutical, and Boehringer Ingelheim Japan. Dr. Azuma received honoraria and grants from Ono Pharmaceutical, and honoraria from Bristol‐Myers Squibb, AstraZeneca, and Chugai Pharmaceutical. Dr. Hayashi received honoraria from AstraZeneca, Boehringer Ingelheim Japan, Bristol‐Myers Squibb, Chugai Pharmaceutical, Eli Lilly Japan, MSD, Ono Pharmaceutical, Pfizer Japan, Shanghai Haihe Biopharm and Taiho Pharmaceutical, and research funding from AstraZeneca, Boehringer Ingelheim Japan, and Ono Pharmaceutical. Dr. Haratani received grants and honoraria from AstraZeneca, honoraria from Ono Pharmaceutical, AS ONE Corporation, Bristol‐Myers Squibb, Chugai Pharmaceutical, MSD, and Pfizer Japan. Dr. Tanaka received honoraria from AstraZeneca, Merck Serono, Bristol‐Myers Squibb, MSD, and Boehringer Ingelheim Japan. Dr. Yonesaka received grants from Daiichi‐Sankyo. Dr. Ishii received grants and honoraria from Boehringer Ingelheim Japan, honoraria from Ono Pharmaceutical, Chugai Pharmaceutical, AstraZeneca and MSD. Dr. Nakagawa received grants and honoraria from MSD, Eli Lilly Japan, Bristol‐Myers Squibb, Taiho Pharmaceutical, Ono Pharmaceutical, Chugai Pharmaceutical, AstraZeneca, Astellas Pharma, Novartis Pharma, Nippon Boehringer Ingelheim, Pfizer Japan, Takeda Pharmaceutical, SymBio Pharmaceuticals, Daiichi Sankyo, grants from Merck Serono, ICON Japan, PAREXEL International, IQVIA Services JAPAN, A2 Healthcare, AbbVie, EP‐CRSU, Linical, Otsuka Pharmaceutical, EPS International, Quintiles, CMIC Shift Zero, Eisai, Kissei Pharmaceutical, Kyowa Hakko Kirin, EPS, Bayer Yakuhin, inVentiv Health Japan, GRITSONE ONCOLOGY, GlaxoSmithKline, Yakult Honsha, Covance, honoraria from KYORIN Pharmaceutical, CareNet, Nichi‐Iko Pharmaceutical, Hisamitsu Pharmaceutical, YODOSHA, Clinical Trial, MEDICUS SHUPPAN,Publishers, from AYUMI Pharmaceutical, Nikkei Business Publications, Thermo Fisher Scientific, NANZANDO, Medical Review, YOMIURI TELECASTING, and Reno. Medical. The other authors declare no potential financial conflicts of interest.

## Supporting information


**Figure S1** Relation Between H‐Scores for AXL and GAS6 mRNA in Tumor Tissue of NSCLC Patients with *EGFR* Activating Mutations.
**Figure S2** Relation Between Circulating Concentrations of AXL or GAS6 and That of CRP for NSCLC Patients with *EGFR* Activating Mutations.
