# Supplementary figures and images for: Tumor tissue and plasma levels of AXL and GAS6 before and after tyrosine kinase inhibitor treatment in EGFR‐mutated non–small cell lung cancer
Source: Thorac Cancer. 2019 Aug 16;10(10):1928–35. doi: 10.1111/1759-7714.13166 (PMC6775020; doi:10.1111/1759-7714.13166)

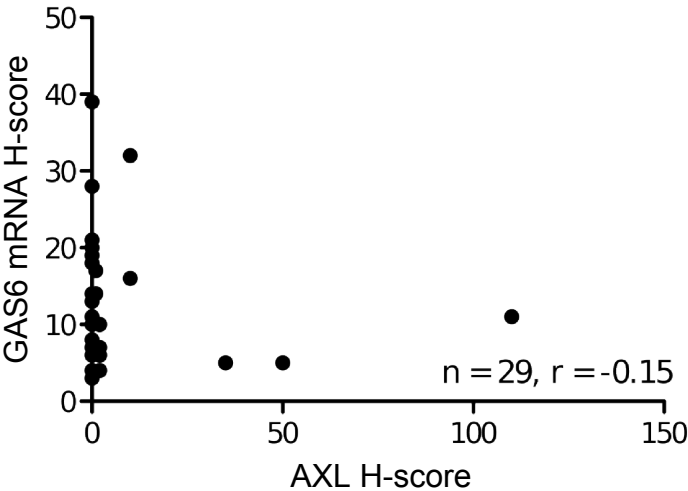

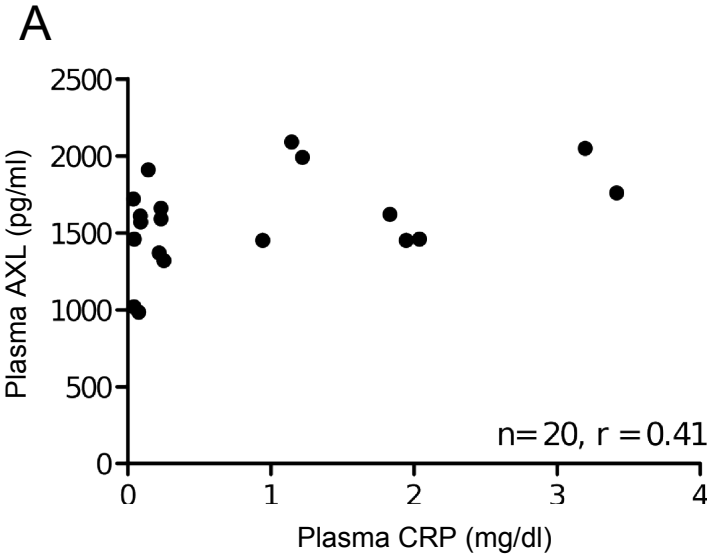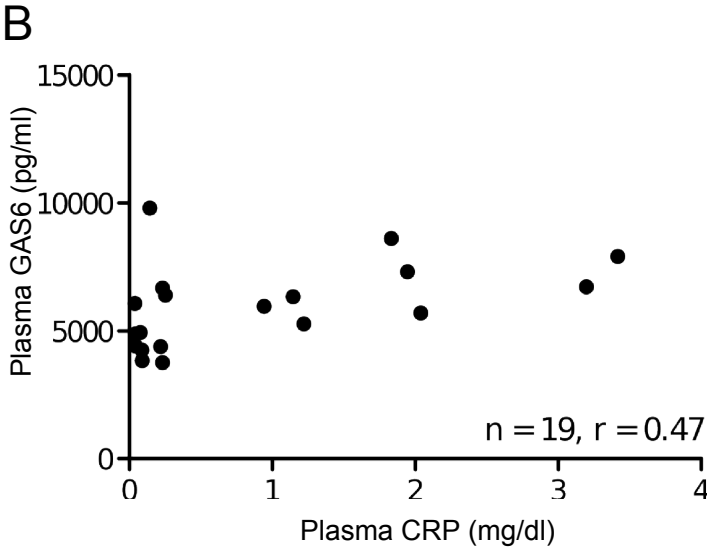

Supplement: Supplementary file 1 — Figure S1 Relation Between H‐Scores for AXL and GAS6 mRNA in Tumor Tissue of NSCLC Patients with EGFR Activating Mutations. Figure S2 Relation Between Circulating Concentrations of AXL or GAS6 and That of CRP for NSCLC Patients with EGFR Activating Mutations. [file TCA-10-1928-s001.pdf]
